# The Association Between Glucagon-like Peptide-1 Receptor Agonists and Clinical Outcomes in Patients with Thoracic Aortic Aneurysm

**DOI:** 10.3390/diagnostics16111742

**Published:** 2026-06-05

**Authors:** Mohammad Alaa Raslan, Hussein Abdul Nabi, Luke Dreher, Soad Al Osta, Vinicius De Sousa Barzon Serra, Chadi Ayoub, Hicham El Masry, Fadi E. Shamoun

**Affiliations:** 1 Department of Cardiovascular Medicine, Mayo Clinic Arizona, 5777 E Mayo Blvd, Phoenix, AZ 85054, USA; raslan.mohammedalaa@mayo.edu (M.A.R.); abdulnabi.hussein@mayo.edu (H.A.N.); dreher.luke@mayo.edu (L.D.); desousabarzonserra.vinicius@mayo.edu (V.D.S.B.S.); ayoub.chadi@mayo.edu (C.A.); elmasry.hicham@mayo.edu (H.E.M.); 2 Department of Internal Medicine, University of Michigan Health—Sparrow, 1215 E Michigan Ave, Lansing, MI 48912, USA; 3 Department of Internal Medicine, MedStar Health Georgetown University, Baltimore, MD 21218, USA; soad.alosta@medstar.net

**Keywords:** glucagon-like peptide-1 receptor agonists, thoracic aortic aneurysm, thoracic aortic dissection, mortality, aorta, cardiovascular outcomes

## Abstract

**Background/Objectives:** Thoracic aortic aneurysm (TAA) often results from structural degeneration of the aortic wall. Traditional management focuses on hemodynamic control using beta-blockers (BB) and angiotensin receptor blockers (ARBs). Glucagon-like peptide-1 receptor agonists (GLP-1 RAs), originally developed for diabetes and weight management, may offer additional vascular protective benefits through anti-inflammatory, antioxidative, and matrix-stabilizing mechanisms. However, their role in reducing mortality and thoracic aortic dissection (TAD) risk in patients with TAA has not been evaluated in human populations. In this study, we aimed to assess the association between GLP-1 RA use and the risks of mortality and thoracic aortic dissection. **Methods:** We conducted a retrospective cohort study of adults diagnosed with TAA between 2018 and 2024 across three Mayo Clinic sites. Patients receiving GLP-1 RAs were compared with non-users using 1:1 propensity score matching. Outcomes included all-cause mortality, cardiovascular mortality, and incident TAD. Kaplan–Meier and Cox proportional hazards analyses were performed. **Results:** A total of 32,279 patients with TAA, with a median age of 68.0 [IQR: 59.0–76.0] and 70.7% male, were included in a 1:1 propensity score match. This yielded a balanced cohort of 588 GLP-1 RA users and 588 non-users. Through a median follow-up of 4.1 (2.2, 5.9) years, GLP-1 RA use was associated with significantly lower 5-year cumulative incidence of all-cause mortality (5.0% vs. 14.5%, HR: 0.31; 95% CI: 0.19–0.50; *p* < 0.001), cardiovascular mortality (1.9% vs. 5.5%, HR: 0.30; 95% CI: 0.13–0.70; *p* = 0.005), and TAD (0.9% vs. 4.0%, HR: 0.19; 95% CI: 0.06–0.60; *p* < 0.0004). **Conclusions:** GLP-1 RAs are associated with reduced incidence of all-cause mortality, cardiovascular mortality, and TAD incidence in patients with TAA. Prospective studies are needed to confirm these findings and evaluate effects on aneurysm progression.

## 1. Introduction

Thoracic aortic aneurysm (TAA) is characterized by expansile dilation of the thoracic aorta, posing a risk for life-threatening complications such as thoracic aortic dissection (TAD). The prevalence of TAA is estimated to be approximately 5.9 per 100,000 person-years, although this may be underestimated due to its often asymptomatic nature and increased detection with advanced imaging modalities [1]. The pathogenesis of TAA involves medial degeneration, elastic fiber fragmentation, vascular smooth muscle cell dysfunction, and extracellular matrix disruption, rendering the aortic wall susceptible to progressive weakening and dilation [1,2]. In addition, chronic inflammation and oxidative stress play key roles in aneurysm formation and progression [2,3,4,5]. Progression to TAD is often catastrophic, with over 50% of affected patients dying within the first 48 h after onset [6]. Given its sudden onset and high mortality, identifying modifiable predictors and effective preventive pharmacologic interventions remains critical.

Traditionally, medical therapies have aimed to reduce hemodynamic stress on the dilated aorta. Beta-blockers lower aortic wall shear stress and heart rate, thereby reducing dissection risk—particularly in patients with Marfan syndrome and related inherited aortopathies [7]. Agents targeting the renin–angiotensin system have also been utilized to mitigate adverse aortic remodeling, although evidence for benefit in non-syndromic TAA remains limited [8]. Current guidelines emphasize strict blood pressure control and surveillance imaging but acknowledge the absence of pharmacologic therapies proven to directly prevent aneurysm progression or dissection in routine clinical practice [9]. This highlights a critical gap in the management of TAA. Addressing this gap is particularly important given the increasing detection of TAA through widespread use of advanced imaging modalities and the aging population.

Beyond standard risk factor modification, there is increasing recognition that thoracic aortic aneurysm represents a complex, multifactorial disease driven by interactions between hemodynamic stress, genetic susceptibility, and maladaptive vascular remodeling [2,3,4,5]. While guideline-directed therapies such as beta-blockers and renin–angiotensin system inhibitors aim to reduce wall stress, they do not directly address the molecular mechanisms underlying aneurysm progression, including extracellular matrix degradation, inflammation, and oxidative stress [3,8,9]. As a result, pharmacologic strategies that target these biological pathways remain an area of significant unmet clinical need.

In recent years, there has been growing interest in repurposing cardiometabolic therapies with pleiotropic vascular effects for structural cardiovascular diseases. Among these, glucagon-like peptide-1 receptor agonists (GLP-1 RAs) have emerged as promising candidates due to their demonstrated benefits across a spectrum of cardiovascular conditions [10,11,12,13,14]. In addition to glycemic control, these agents have been shown to reduce systemic inflammation, improve endothelial function, and modulate oxidative stress pathways, all of which are implicated in the pathophysiology of aortic aneurysm formation and progression [15,16,17,18,19]. However, despite strong mechanistic rationale and supportive preclinical data, clinical evidence evaluating their role in aortic disease remains lacking.

In addition to these established mechanisms, emerging evidence suggests that thoracic aortic aneurysm represents a systemic vascular disorder rather than an isolated structural abnormality. Increasing data highlight the contribution of chronic low-grade inflammation, endothelial dysfunction, and metabolic dysregulation to aneurysm development and progression [2,3,4,5]. These processes overlap significantly with pathways implicated in atherosclerosis and cardiometabolic disease, suggesting potential shared therapeutic targets [16,17,18,19].

Despite advances in imaging and surgical management, pharmacologic therapy for TAA remains largely supportive, with no agents definitively shown to alter the natural history of the disease [9]. This has led to growing interest in repurposing therapies with known cardiovascular benefits for potential use in structural vascular disease. Identifying therapies that can simultaneously address systemic cardiovascular risk and local vascular pathology represents a key unmet need in contemporary TAA management.

Glucagon-like peptide-1 receptor agonists (GLP-1 RAs), originally developed as cardio-metabolic medications, have demonstrated broader cardiovascular and vascular protective effects beyond glycemic control [10,11,12,13,14]. These agents have been shown to reduce major adverse cardiovascular events, including myocardial infarction, stroke, and cardiovascular mortality [11,12,13,14]. Their effects appear to extend beyond glucose lowering and include improvements in endothelial function, reductions in oxidative stress, and attenuation of inflammatory signaling pathways [15,16,17,18,19]. Furthermore, GLP-1 receptors are expressed in vascular tissues, supporting a potential direct role in modulating vascular homeostasis [20].

Preclinical studies support this hypothesis. GLP-1 RA therapy has been shown to attenuate aneurysm formation and progression through reduction in oxidative stress, suppression of macrophage-mediated inflammation, and preservation of extracellular matrix integrity [21,22]. These mechanisms are central to aneurysm pathophysiology, including degradation of elastin and collagen mediated by matrix metalloproteinases [23]. Despite these findings, no human study to date has evaluated the association between GLP-1 RA use and clinical outcomes in TAA. Therefore, we conducted the present study to evaluate the association between GLP-1 RA use and clinical outcomes in patients with TAA.

## 2. Materials and Methods

### 2.1. Study Population

A retrospective cohort analysis of patients (age ≥ 18 years) diagnosed with thoracic aortic aneurysm (TAA) between 2018 and 2024, across three Mayo Clinic sites (Rochester, Minnesota; Jacksonville, Florida; and Phoenix, Arizona) was conducted. The study was approved by the institutional review board (IRB No. 24-004593), with waiver of informed consent.

Patients were identified using International Classification of Diseases, Tenth Revision (ICD-10) codes validated in prior studies. To ensure diagnostic accuracy, an electronic data extraction tool was used to confirm TAA diagnosis via computed tomography (CT) imaging or echocardiography. Patients without confirmatory CT angiography or echocardiograms were excluded from the study. In patients with echocardiographic data available, baseline measurements of the sinus of Valsalva (SoV) and mid-ascending aorta were collected from the first available echocardiogram and follow-up studies. At Mayo Clinic, thoracic aortic aneurysm severity is assessed using individualized reference values adjusted for sex, body surface area (BSA), and age rather than a single universal diameter threshold. Accordingly, aortic dimensions were interpreted in the context of patient-specific expected reference values, as a given absolute aortic diameter may represent different degrees of pathological dilation across individuals. A centralized database was created using electronic medical records (EMR) to extract demographic data, clinical characteristics, and comorbidities retrospectively.

Medication data were retrieved from the EMR. This included beta-blockers (BB), angiotensin receptor blockers (ARBs), and GLP-1 receptor agonists, including semaglutide, liraglutide, dulaglutide, exenatide, and tirzepatide. A patient was deemed to be taking a medication if it had been prescribed and continued for at least one year after the TAA diagnosis. The cohort was then divided into two groups: those treated with GLP-1 RAs and those not receiving this therapy.

Baseline variables included:

- Demographics (age, sex, race);
- Cardiovascular risk factors (hypertension, diabetes, smoking);
- Structural conditions (bicuspid valve, connective tissue disorders);
- Medications (beta-blockers, ACEi/ARB, statins).

### 2.2. Propensity Score Matching

To reduce confounding, 1:1 propensity score matching was performed using nearest-neighbor matching with a caliper of 0.1 of the logit of the propensity score.

### 2.3. Study Outcomes

The primary outcome was to evaluate all-cause mortality in TAA patients. Secondary outcomes were the incidence of aortic dissection and cardiovascular mortality. For patients identified with an aortic dissection diagnosis or a recorded death, manual chart review confirmed the events and documented the date of occurrence.

### 2.4. Statistical Analysis

Baseline characteristics were compared between the GLP-1 RA users and non-users using Student’s *t*-test or non-parametric tests for continuous variables, depending on distribution, and chi-square or Fisher exact tests for categorical variables. Continuous variables were reported as mean ± standard deviation (SD) or median with interquartile range (IQR), and categorical variables as frequencies and percentages. To reduce potential confounders, propensity score matching was performed using a 1:1 nearest-neighbor algorithm without replacement and a caliper of 0.1 times the SD of the logit of the propensity scores.

Cumulative incidences of all-cause mortality, cardiovascular mortality, and thoracic aortic dissection were compared using Kaplan–Meier survival curves and the log-rank test. A univariable Cox proportional hazards model evaluated the association between GLP-1 RA use and clinical outcomes. Time zero was defined as the date of TAA diagnosis for non-users, and the GLP-1 RA prescription date for users. Patients were censored at whichever occurred first: the outcome of interest, death, or their last clinical encounter. Follow up was limited to five years. Statistical significance was set at a two-tailed *p* < 0.05, and all analyses were performed with R version 4.3 (R Foundation for Statistical Computing). This approach ensured consistent follow-up across both cohorts while minimizing bias related to differential follow-up duration. However, because time zero differed between GLP-1 RA users and non-users, the possibility of immortal time bias cannot be completely excluded and should be considered when interpreting the findings.

To further ensure robustness of the findings, standardized mean differences (SMDs) were used to assess balance between matched cohorts, with values < 0.1 considered indicative of adequate covariate balance. Although propensity score matching was employed to reduce confounding, residual confounding cannot be fully excluded given the observational design. Additionally, sensitivity analyses evaluating the consistency of findings across subgroups defined by diabetes status and baseline cardiovascular risk factors may further strengthen causal inference; however, such analyses were beyond the scope of the present study.

## 3. Results

**Figure 1:**
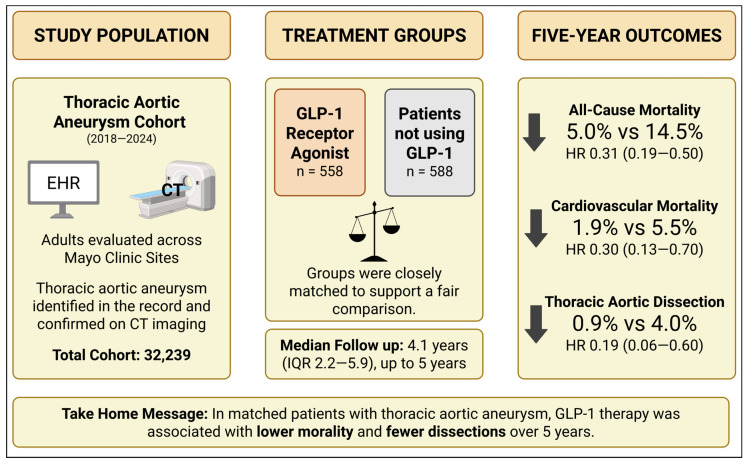
GLP-1 Receptor Agonist Therapy and Five-Year Outcomes in Thoracic Aortic Aneurysm. Study design and key outcomes among adults with thoracic aortic aneurysm evaluated across Mayo Clinic sites (2018–2024). Patients receiving GLP-1 receptor agonist therapy (*n* = 588) were compared with a closely matched group of patients not using GLP-1 therapy (*n* = 588) with median follow-up of 4.1 years (IQR 2.2–5.9), up to 5 years. Over 5 years, GLP-1 therapy was associated with lower all-cause mortality (5.0% vs. 14.5%; HR 0.31, 95% CI 0.19–0.50), lower cardiovascular mortality (1.9% vs. 5.5%; HR 0.30, 95% CI 0.13–0.70), and fewer thoracic aortic dissections (0.9% vs. 4.0%; HR 0.19, 95% CI 0.06–0.60). “Created in BioRender. Dreher, L. (2026) https://BioRender.com/pc3vn74 (accessed on 4 March 2026)”. Abbreviations: CT, computed tomography; EHR, electronic health record; GLP-1, glucagon-like peptide-1; HR, hazard ratio; IQR, interquartile range.

Of the 32,279 patients with TAA identified in the full cohort, 1:1 propensity-score matching produced 588 GLP-1 RA users and 588 non-users, with all matched variables exhibiting standardized mean differences < 0.01. Baseline characteristics of the overall and matched cohorts are summarized in Table 1. The median age was 63.0 years (IQR 56.0–69.0). In total, 795 patients (67.6%) were male, and 1115 (94.8%) self-identified as White. Among the GLP-1 RA users, 434 (73.8%) were prescribed the agent for type 2 diabetes mellitus and 154 (26.2%) for weight loss. Figure 1 presents the central illustration of the study and summarizes the key findings.

**Figure 2:**
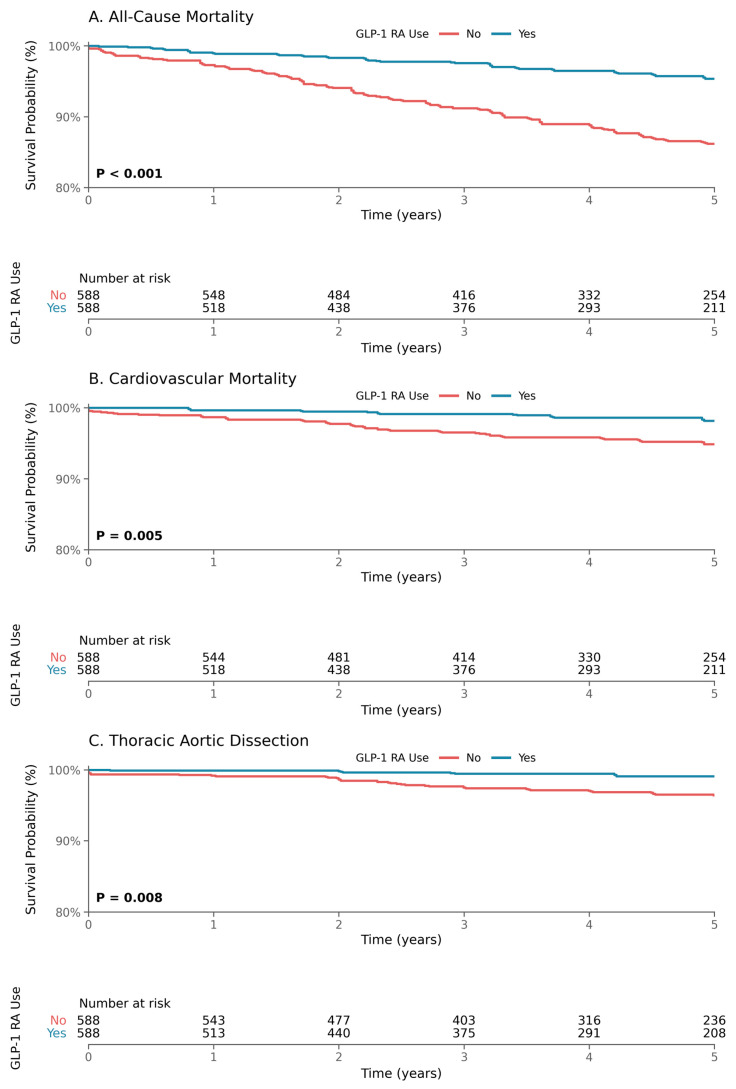
Kaplan–Meier survival curves comparing survival-free probability from (**A**) all-cause mortality, (**B**) cardiovascular mortality, (**C**) thoracic aortic dissection between patients who were using glucagon-like peptide 1 agonists and patients who were not.

Over five years, the cumulative incidence of all-cause mortality, cardiovascular mortality, and thoracic aortic dissection (TAD) was 10.1%, 3.7%, and 2.5%, respectively. GLP-1 RA users had significantly lower 5-year incidence of all-cause mortality compared to non-users (5.0% vs. 14.5%; log-rank *p* < 0.001) (Figure 2A). Univariable Cox regression analysis confirmed the reduced risk of all-cause mortality at five years among GLP-1 RA users (hazard ratio [HR] 0.31, 95% CI 0.19–0.50; *p* < 0.001) (Table 2). These findings correspond to an absolute risk reduction of approximately 9.5% in all-cause mortality over five years, highlighting the potential clinical relevance of the observed association.

The five-year cumulative incidence of cardiovascular mortality was significantly lower in GLP-1 RA users compared to non-users (1.9% vs. 5.5%; log Rank *p* = 0.005) (Figure 2B). Univariable Cox regression analysis confirmed a reduced risk of cardiovascular mortality at 5 years in GLP-1 RA users (HR 0.30, 95% CI 0.13–0.70; *p* = 0.005) (Table 2).

The five-year cumulative incidence of TAD was also lower among GLP-1 RA users, with a statistically significant difference compared to non-users (0.9% vs. 4.0%; *p* = 0.004; Figure 2C). Univariable Cox regression analysis further confirmed a significantly reduced five-year risk of TAD in GLP-1 RA users (HR 0.19, 95% CI 0.06–0.60; *p* = 0.004) (Table 2).

Baseline echocardiographic aortic dimensions were comparable between groups. Among GLP-1 RA users, the mean enlarged mid-ascending aortic diameter at the first echocardiogram was 43.38 ± 3.42 mm, while the mean enlarged sinus of Valsalva (SoV) diameter was 44.44 ± 3.05 mm. At the last available echocardiogram, the mean enlarged mid-ascending aortic diameter was 44.15 ± 3.39 mm and the mean enlarged SoV diameter was 44.95 ± 3.15 mm. Among non-GLP-1 RA users, the mean enlarged mid-ascending aortic diameter at the first echocardiogram was 44.35 ± 3.16 mm, while the mean enlarged SoV diameter was 45.67 ± 3.26 mm. At the last available echocardiogram, the mean enlarged mid-ascending aortic diameter was 46.00 ± 3.77 mm.

## 4. Discussion

Thoracic aortic aneurysm remains a clinically significant condition due to its potential progression to life-threatening complications such as thoracic aortic dissection, which is associated with substantial early mortality and morbidity. Despite advances in surgical and endovascular therapies, pharmacologic management remains limited and is primarily focused on reducing hemodynamic stress rather than directly modifying disease biology or progression. Current guideline-directed therapy emphasizes blood pressure control and the use of agents such as beta-blockers and renin–angiotensin system inhibitors, yet no pharmacologic intervention has been definitively shown to prevent aneurysm progression or dissection in routine clinical practice [9,20]. In this multi-center retrospective cohort study, we demonstrate that GLP-1 RA use is associated with significantly lower risks of all-cause mortality, cardiovascular mortality, and TAD in patients with TAA. To our knowledge, this represents the first clinical study evaluating this association.

From a clinical perspective, the magnitude of association observed in this study is particularly notable and clinically meaningful. The relative risk reductions in both all-cause and cardiovascular mortality, as well as thoracic aortic dissection, suggest that GLP-1 receptor agonists may influence not only systemic cardiovascular risk but also disease-specific outcomes related to aortic pathology across a broad range of patients. While causality cannot be established, the consistency of effect across multiple clinically relevant endpoints strengthens the plausibility of a true biological relationship. These findings may have important clinical implications for patients with TAA, particularly those with coexisting metabolic disease, where GLP-1 RAs are already commonly prescribed [10,11,12,13,14].

GLP-1 RA use was associated with substantial reductions in mortality and dissection risk over a five-year follow-up period. These findings are particularly relevant when considered alongside traditional therapies, which primarily target aortic wall stress rather than the underlying structural and molecular mechanisms of aneurysm progression [8,9]. While beta-blockers have demonstrated benefit in specific populations such as Marfan syndrome, their effect in broader TAA populations remains less clearly defined. The observed associations in our study suggest that GLP-1 RAs may provide additional benefit through mechanisms beyond hemodynamic modulation.

Importantly, the association between GLP-1 RA use and improved outcomes was observed across patients with and without diabetes, supporting the concept that their effects extend beyond glycemic control. This is consistent with findings from large cardiovascular outcome trials demonstrating reductions in major adverse cardiovascular events and mortality with GLP-1 RA therapy [11,12,13,14]. The SELECT trial further supports this concept, demonstrating cardiovascular benefit in individuals with obesity without diabetes [14].

Although no prior human studies have evaluated GLP-1 RAs specifically in TAA, accumulating experimental and translational evidence provides a strong mechanistic rationale for their potential benefit. Preclinical studies have demonstrated that GLP-1 RA therapy reduces oxidative stress, suppresses macrophage-mediated inflammation, and attenuates vascular injury in models of aortic aneurysm [21,22]. These processes are central to aneurysm development and progression, including extracellular matrix degradation, inflammatory activation, and vascular smooth muscle cell dysfunction [2,3,4,5]. Genetic susceptibility and clinical heterogeneity further contribute to disease progression and variability in outcomes across patient populations [24].

The underlying mechanisms by which GLP-1 RAs may influence aortic disease are likely multifactorial. GLP-1 receptors are expressed in human vascular tissues, including the aorta, allowing for direct effects on vascular structure and function [20]. Activation of GLP-1 signaling has been shown to improve endothelial function, increase nitric oxide bioavailability, and reduce oxidative stress, thereby promoting vascular homeostasis [15,16,17,18]. In addition, GLP-1 RAs exert anti-inflammatory effects by modulating macrophage activity and reducing pro-inflammatory cytokine signaling [17,19]. Emerging data also support broader anti-atherosclerotic and vascular protective effects of GLP-1 receptor signaling in human vascular disease [25].

Extracellular matrix degradation is a hallmark of aneurysm pathophysiology and is largely mediated by matrix metalloproteinases (MMPs), particularly MMP-2 and MMP-9 [3,23]. Experimental studies suggest that GLP-1 RA therapy may indirectly suppress MMP activity through anti-inflammatory pathways, thereby preserving elastin and collagen integrity within the aortic wall [21,22]. In addition to these direct vascular effects, GLP-1 RAs improve systemic cardiovascular risk factors, including blood pressure, weight, and metabolic parameters [26,27]. Even modest reductions in blood pressure may reduce aortic wall stress over time. Recent evidence further highlights the central role of inflammation and oxidative stress in driving aneurysm progression and matrix remodeling [28,29].

Beyond these mechanistic considerations, the potential impact of GLP-1 receptor agonists on vascular remodeling warrants further attention. Vascular smooth muscle cell phenotype switching, a key driver of aneurysm progression, is influenced by inflammatory and metabolic signaling pathways that may be modulated by GLP-1 receptor activation [2,3,4,5]. By promoting a more stable vascular phenotype and reducing pro-inflammatory signaling, GLP-1 receptor agonists may contribute to preservation of aortic wall integrity over time [15,16,17,18,19]. These effects, while not directly measured in the present study, provide a biologically plausible framework supporting the observed clinical associations.

These findings have important clinical implications. In particular, the observed associations may inform future strategies aimed at early intervention and risk modification in patients with asymptomatic disease. GLP-1 RAs may represent a novel pharmacologic approach for risk modification in patients with TAA, addressing a key gap in current management strategies [9,20]. Given their established safety profile and widespread use, they may be readily repurposed pending further validation. Thoracic aortic aneurysm remains a clinically heterogeneous condition with variable presentation, risk profiles, and management considerations.

Importantly, the real-world applicability of these findings enhances their clinical relevance. GLP-1 receptor agonists are already widely prescribed in patients with type 2 diabetes and obesity, populations that frequently overlap with individuals at risk for thoracic aortic aneurysm [11,12,13,14]. The observed associations therefore raise the possibility that existing therapies may confer previously unrecognized benefits in this population without the need for novel drug development. This paradigm of therapeutic repurposing is particularly attractive in conditions such as TAA, where effective medical therapies remain limited [9].

The magnitude of risk reduction observed in this study appears comparable to or greater than that reported with traditional therapies such as beta-blockers and renin–angiotensin system inhibitors, which primarily target hemodynamic stress rather than vascular remodeling. These findings suggest that GLP-1 RAs may exert complementary effects by modulating underlying vascular biology. Given their established safety profile and widespread use, these agents may offer a readily translatable therapeutic strategy in patients with TAA, particularly those with coexisting cardiometabolic disease [27].

In the broader context of emerging therapies for aortic disease, several pharmacologic strategies have been investigated with varying degrees of success. Agents targeting the renin–angiotensin system, anti-inflammatory pathways, and matrix metalloproteinase activity have shown promise in preclinical and select clinical studies, yet translation into consistent clinical benefit has been limited [3,8,9,23]. In contrast, GLP-1 receptor agonists offer a unique therapeutic profile by simultaneously targeting multiple pathways implicated in aneurysm pathophysiology, including inflammation, oxidative stress, and metabolic dysfunction [15,16,17,18,19]. This multimodal mechanism of action may partly explain the magnitude of association observed in the present study and suggests a potential advantage over therapies that target single pathways in isolation.

From a practical standpoint, these findings raise important considerations regarding the integration of GLP-1 receptor agonists into the management of patients with thoracic aortic aneurysm. Current therapeutic strategies are largely limited to risk factor modification and surveillance, with pharmacologic therapy primarily targeting blood pressure reduction. In this context, GLP-1 receptor agonists may represent a complementary strategy that addresses both systemic cardiovascular risk and underlying vascular biology. Their use may be particularly relevant in patients with concomitant metabolic disease, where they are already indicated, potentially offering dual benefit without additional therapeutic burden.

From a clinical decision-making perspective, the potential role of GLP-1 receptor agonists in TAA management raises important considerations regarding patient selection and timing of therapy initiation. Patients with early-stage aneurysmal disease, in whom structural progression may still be modifiable, could represent an ideal target population for intervention. Additionally, individuals with concomitant cardiometabolic conditions such as obesity, insulin resistance, or type 2 diabetes may derive compounded benefit from GLP-1 receptor agonist therapy through simultaneous modification of systemic and vascular risk factors [11,12,13,14,27]. Conversely, in patients with advanced aneurysmal disease approaching surgical thresholds, the relative impact of pharmacologic therapy may be more limited [9]. These considerations highlight the need for a nuanced, patient-centered approach when evaluating the potential incorporation of GLP-1 receptor agonists into clinical practice.

In addition, these findings may have implications for risk stratification in TAA. Identifying patients at higher risk of adverse outcomes remains a clinical challenge, particularly in those without syndromic disease. If validated, GLP-1 receptor agonist use could potentially serve as a marker of modifiable risk or as part of a broader therapeutic strategy aimed at reducing progression to dissection. This concept aligns with the evolving paradigm of precision medicine, where treatment strategies are increasingly tailored to both systemic and disease-specific risk profiles.

Thoracic aortic aneurysm is a clinically heterogeneous condition, with variability in etiology, rate of progression, and risk of adverse outcomes [24]. Patients with genetic syndromes, bicuspid aortic valve disease, and degenerative aneurysms represent distinct subgroups with differing pathophysiological mechanisms [2,3,4,5,24]. The consistent association observed in this study across a broad patient population suggests that GLP-1 receptor agonists may exert effects that are not limited to a single disease subtype. However, further investigation is needed to determine whether certain patient populations derive greater benefit than others.

Several important limitations should be acknowledged. First, the retrospective observational design precludes causal inference, and residual confounding may persist despite propensity score matching. In particular, confounding by indication remains a concern, as patients receiving GLP-1 RAs may differ systematically from non-users in ways not fully captured by measured covariates. Second, the definition of GLP-1 receptor agonist exposure based on prescription data and continuation for at least one year may introduce selection bias and may not fully reflect medication adherence, discontinuation, dose adjustments, or treatment changes over time. Patients who remained on therapy for longer durations may also represent a healthier or more closely monitored population, potentially influencing observed outcomes. Because medication exposure was determined retrospectively from electronic medical records, residual exposure misclassification cannot be completely excluded. Third, the use of different index time definitions between exposure groups may introduce immortal time bias, potentially influencing the observed associations. Although this approach was selected to better reflect real-world treatment exposure in a retrospective observational cohort, residual immortal time bias may still persist despite propensity score matching and careful cohort construction. Therefore, the observed associations should be interpreted cautiously and considered hypothesis-generating rather than definitive evidence of causality. Prospective randomized controlled studies will ultimately be required to confirm the potential effects of GLP-1 receptor agonists on thoracic aortic aneurysm outcomes. Fourth, the lack of longitudinal imaging data limits assessment of aneurysm growth and structural progression, which are key intermediate outcomes in TAA [2,3,4,5]. Finally, reliance on administrative coding and electronic health record data introduces the potential for misclassification, although efforts were made to validate diagnoses using imaging confirmation [30].

In addition to their direct vascular effects, the potential influence of GLP-1 receptor agonists on longitudinal disease trajectories warrants consideration. Thoracic aortic aneurysm progression is often characterized by slow, subclinical expansion punctuated by acute events such as dissection. Therapies that can modulate underlying inflammatory and metabolic pathways may therefore have cumulative benefits over time, even if short-term structural changes are not immediately apparent. This concept is supported by the broader cardiovascular literature, where GLP-1 receptor agonists have demonstrated sustained reductions in adverse events over extended follow-up periods [11,12,13,14,15,16,17,18,19]. As such, the benefits observed in this study may reflect both acute and chronic effects on vascular stability, further supporting their potential role in modifying the natural history of aortic disease.

Future studies should focus on prospective randomized trials designed to evaluate the effect of GLP-1 receptor agonists on aneurysm growth, aortic remodeling, and clinical outcomes in patients with thoracic aortic aneurysm. Such studies should incorporate serial imaging to assess structural progression and explore potential differential effects across patient subgroups, including those with and without diabetes. In addition, mechanistic studies integrating biomarkers of inflammation, oxidative stress, and extracellular matrix remodeling may further clarify the biological pathways underlying the observed associations.

## 5. Conclusions

GLP-1 receptor agonist use is associated with significantly lower risks of all-cause mortality, cardiovascular mortality, and thoracic aortic dissection in patients with thoracic aortic aneurysm. These findings suggest a potential role for GLP-1 receptor agonists beyond cardiometabolic disease, extending into structural vascular pathology. In the absence of effective pharmacologic therapies targeting aneurysm progression, these results highlight a promising avenue for therapeutic repurposing. Prospective studies are warranted to confirm these associations and to further define the role of GLP-1 receptor agonists in modifying disease progression, aortic remodeling, and long-term clinical outcomes in this high-risk population. Such studies will be essential to determine optimal patient selection, timing of therapy initiation, and the potential integration of these agents into existing treatment paradigms.

## Figures and Tables

**Table 1:** Baseline Characteristics of the overall and matched cohort with a comparison between patients receiving GLP-1 RA therapy and patients who were not.

	Before 1:1 Propensity Score Matching				After 1:1 Propensity Score Matching		
Characteristics	Non-GLP-1 RA Users (*N* = 31,687)	GLP-1 RA Users (*N* = 592)	SMD	*p* Value	Non-GLP-1 RA Users (*N* = 588)	GLP-1 RA Users (*N* = 588)	SMD
Age (at diagnosis), years	68 (59, 76)	63 (56, 69)	−0.278	<0.001	63 (55, 69)	63 (56, 69)	−0.095
Male sex	22,436 (70.8%)	390 (65.9%)	0.045	0.009	405 (68.9%)	390 (66.3%)	0.010
Tobacco use	12,854 (40.6%)	273 (46.1%)	0.109	0.006	267 (45.4%)	270 (45.9%)	−0.017
BMI > 25	8884 (28%)	448 (75.7%)	0.895	<0.001	445 (75.7%)	444 (75.5%)	−0.061
Comorbidities							
HTN	21,776 (68.7%)	517 (87.3%)	0.343	<0.001	524 (89.1%)	513 (87.2%)	−0.069
Diabetes Mellitus	6043 (19.1%)	378 (63.9%)	1.033	<0.001	370 (62.9%)	374 (63.6%)	−0.042
Connective tissue disease syndromes **	768 (2.4%)	7 (1.2%)	−0.171	0.051	8 (1.4%)	7 (1.2%)	0.021
Bicuspid aortic valve	4725 (14.9%)	84 (14.2%)	−0.077	0.625	81 (13.8%)	84 (14.3%)	0.032
Heart failure	12,821 (40.5%)	290 (49%)	0.150	<0.001	292 (49.7%)	289 (49.1%)	0.035
ASCVD **	10,118 (31.9%)	198 (33.4%)	0.091	0.434	197 (33.5%)	198 (33.7%)	−0.055
Medications							
Statins	17,145 (54.1%)	354 (59.8%)	−0.320	0.006	356 (60.5%)	353 (60.0%)	−0.093
Angiotensin-converting enzyme inhibitors/angiotensin II receptor blockers	14,885 (47%)	342 (57.8%)	−0.242	<0.001	331 (56.3%)	340 (57.8%)	−0.080
β-Blockers	19,848 (62.6%)	414 (69.9%)	0.191	<0.001	422 (71.8%)	410 (69.7%)	−0.043

Abbreviations: GLP-1 RAs: glucagon-like peptide 1 receptor agonists; BMI: body mass index; HTN: hypertension; ASCVD: atherosclerotic cardiovascular disease. Continuous variables were reported as median (IQR) or mean ± SD, categorical variables were reported as *N* (%). ** The syndromes represented include Marfan, Loeys-Dietz, and Ehlers-Danlos, while ASCVD comprises myocardial infarction (MI), stroke, and peripheral artery disease (PAD).

**Table 2:** Univariate Cox proportional hazard regression analysis assessing the association between GLP-1 receptor agonists use and clinical outcomes.

Outcomes	HR	*p* Value	95% CI
All-cause mortality	0.31	<0.001	0.19 to 0.51
Cardiovascular mortality	0.30	0.005	0.13 to 0.70
TAD	0.19	0.008	0.06 to 0.60

Abbreviations: HR: hazard ratio; CI: confidence interval; TAD: thoracic aortic dissection.

## Data Availability

The data underlying this article will be shared on reasonable request to the corresponding author.
